# The relationship between academic performance and recreation use among first-year medical students

**DOI:** 10.3402/meo.v20.25105

**Published:** 2015-03-26

**Authors:** Alexander N. Slade, Susan M. Kies

**Affiliations:** Department of Pathology, College of Medicine, University of Illinois at Urbana-Champaign, Urbana, IL, USA

**Keywords:** academic performance, wellness interventions, longitudinal analysis, first-year medical students

## Abstract

**Introduction:**

Self-care activities, including exercise, may be neglected by medical students in response to increasing academic demands. Low levels of exercise among medical students may have ripple effects on patient care and counseling. This study investigates the reciprocal role of recreation use and academic performance among first-year medical students.

**Methods:**

We combined retrospective administrative data from four cohorts of first-year medical students at the University of Illinois at Urbana-Champaign from 2006 to 2010 (*n*=408). We estimated regression models to clarify the role of changes in recreation use before examinations on changes in academic performance, and vice versa.

**Results:**

The use of recreation facilities by first-year medical students was highly skewed. We found that changes in recreation use before an exam were positively associated with changes in exam performance, and vice versa. Students who make large decreases in their recreation use are likely to decrease their exam scores, rather than increase them.

**Discussion:**

Students who make decreases in their recreation, on average, are likely to decrease their exam scores. These findings suggest that medical students may be able to boost their achievement through wellness interventions, even if they are struggling with exams. We find no evidence that decreasing wellness activities will help improve exam performance.

The beneficial effect that physical activity has on health and mortality has been known for decades (1–6). For instance, a 287 kcal increase in energy expenditure per day was found to be associated with an approximately 30% lower risk for mortality in a sample of older Americans (4). Notwithstanding these benefits, medical students sometimes fail to make time investments in their own health, including exercise, in response to the rigors of medical school. Though it has been reported that medical students tend to have relatively high rates of exercise (7–9), it has also been reported that exercise levels can wane as the academic year progresses (10). In fact, in a recent survey of second-year medical students, increased exercise was the leading behavioral change students wanted to make (11). Though the exercise habits of medical students are important for their own physical and mental health, the consequences of poor exercise habits among medical students can extend to their counseling practices in clinical settings (12–15). For instance, physicians that disclose their personal healthy behaviors are more likely to be perceived as believable and motivating about diet and exercise by their patients.

The relationship between academic performance and exercise is an important one, especially in the context of medical students. However, the extent and direction of this relationship has remained ambiguous. A large body of evidence suggests that physical activity is positively associated with cognitive ability (16, 17). While much of the literature has focused on clarifying this relationship in older adults, evidence suggests that this association holds for younger individuals as well (18). Recent research has also tied increases in the utilization of campus recreation (CR) facilities (e.g., gyms) to a higher GPA in undergraduates (19). Additionally, regular exercise, rather than a single bout of exercise, is associated with improvements in cognition, anxiety, and mood in sedentary young adults (20). Several biological observations have been put forth to explain some of the positive associations found between physical activity and cognition. Exercise can lead to an increased proliferation and survival of cells in the hippocampus (21). This observation may be explained, in part, by increased levels of brain-derived neurotrophic factor (BDNF) (22) and neurotrophin granulocyte colony stimulating factor (G-CSF) (23) found in individuals after physical activity. In addition to cognitive performance, increases in physical activity have also been implicated in improvements in mental health outcomes, such as depression and anxiety (24). In this context, physical activity has the potential to offset the stress and other mental health problems that are frequently documented among first-year medical students, especially as the year progresses (9, 25–27).

A possible factor that can influence a students’ decision to engage in recreation activities is their mobile phone. The prevalence of smartphones and/or other handheld computers has exponentially expanded over the past decade. In a 2004 survey of medical students at two US medical schools, 52% of students (28% of preclinical students) reported owning a Personal Data Assistant (28). In a recent study conducted among medical students at the University of Toronto in 2014 (29), nearly all (98%) students surveyed reported using a smartphone. While smartphone use can influence a student's communication and clinical learning, there is a potential for their use to affect personal activities, including recreation use, as well. Lepp et al. (30) discuss that the potential exists for smartphones to enhance physical activity through applications which support individuals both before and during exercise. However, among undergraduate students, they found that increased reported cell phone use is associated with a decreased level of cardiorespiratory fitness. In fact, high levels of cell phone use were found to be correlated with additional sedentary behaviors that may detract from physical activity.

In this study, we merged several sources of administrative data to answer two questions that focus on the relationship between academic performance and physical activity in the first year of medical school. To our knowledge, we are the first study that explores this relationship among medical students. We considered two directions of this relationship. We first considered how changes in the utilization of CR facilities in the 3 weeks prior to an exam predict changes in exam performance. Second, we considered the extent to which changes in exam performance predicts changes in CR use in the 21 days following an exam.

## Methods

### Academic performance

We collected data on 408 traditional first-year medical students over 4 years, from 2006 to 2010. Each year the University of Illinois College Of Medicine at Urbana enrolls approximately 110 first-year (or M-1) students in the traditional pathway.^1^ First-year students take 11 courses, with a total of approximately 46 written examinations throughout the course of the year. Exams are given on approximately 21 different dates throughout the year. While a few exam dates are close together (within 2–3 days of each other), most occur at 2–3 weeks intervals from each other. For each student, a weighted average of exam scores (by the number of items on the exam) is computed separately for each exam date. Exam scores are expressed as the percentage of correct items a student received.

### Campus recreation participation and baseline characteristics

The University of Illinois Campus Recreation system provides athletic- and wellness-related services to undergraduate and graduate students, as well as other members of the Urbana-Champaign campus community. A newly renovated 340,000 square foot facility was opened in 2008, which includes amenities such as a climbing wall, an indoor track, several racquetball courts, a pool, and an instructional kitchen, along with more traditional cardiovascular and resistance training equipment. In addition, CR offers optional accommodations for personal training, group fitness instruction, and nutrition/wellness services. The breadth of recreation-related facilities and services offered give medical students a wide array of resources to become physically active while pursuing their studies. With the University of Illinois at Urbana-Champaign being ranked first by the Princeton Review for the best athletic facilities nationwide in 2015 (31), we believe that our medical students have some of the best recreation offerings in the country.

Membership at all CR facilities was included in the general fees required of all College of Medicine students in order to enroll, which removes monetary cost as a barrier to utilization of CR activities and facilities. However, we did not observe students who choose to purchase alternate gym memberships in the community or exercise outside of a fitness facility. However, the CR system is believed by the University administration to be well-utilized among the general university population. Our study confirmed this intuition among first-year medical students, with the majority of students participating at least once during the academic year, and many frequenting CR facilities at least once per week. The data for CR utilization include the dates and counts for each ‘swipe’ of the student's ID card, which is required for entry into the facility.

Demographic data included sex, age, race, ethnicity, and in-state residency status, which were all measured at the time of matriculation. We also considered undergraduate academic achievement, as measured by MCAT scores and undergraduate GPA. These data were merged together according to a student's University ID number. The data collection techniques and procedures were reviewed and approved by the Institutional Review Board (IRB) at the University of Illinois.

### Statistical models

This study aimed to answer two questions regarding the relationship between recreation facility utilization and academic performance. First, we sought to understand the extent to which the use of CR facilities plays a role in improving examination performance. Second, we were interested in how changes in exam performance may influence students to change their use of CR facilities. A potential issue in addressing these questions is that of simultaneity. Individuals are making choices that affect their CR use and their academic performance at the same time. Likewise, while it is conceivable that increased CR use can affect exam scores, it is plausible that academic performance can also influence CR use. Considering the association of CR use and academic performance for all students pooled over time could potentially mischaracterize these relationships, most likely overstating them. As discussed above, a limitation in our data collection was that we do not observe the wellness or physical fitness activities that students may participate in outside of the CR system. These might include fitness clubs in the community, outdoor activities, or indoor activities at home. Similarly, we are unable to observe the content of the student's visit to a CR facility. Some may use it for an activity like weightlifting, whereas others may use it to participate in a club activity or sport.

Some of these limitations may be partially mitigated by our use of longitudinal data. Longitudinal data allowed us to consider the differences in CR use over time, and how that may potentially be associated with academic performance, which requires looser assumptions than if all the data were cross-sectional. We made the assumption that individuals are likely to use the CR facilities in the same manner over time across their visits, in that individuals, who, for instance, are members of a community fitness club, would be unlikely to start using CR facilities *de novo*. Even if students were to change their mode or location of regular physical activity across the year, we hypothesized that the changes would be unlikely to be related to their academic performance. Furthermore, it is possible that students may continually use CR facilities without exercising, such that the results should be interpreted in terms of ‘recreation utilization’ rather than ‘exercise’.

We estimated two ordinary least squares (OLS) models. In the first model, we predicted changes in academic performance from one exam to the next (as the dependent variable) as a function of changes in the total number of CR visits 21 days before the exam (as the primary independent variable). In addition to the independent variable, we controlled for academic year, sex, age, race, ethnicity, and in-state residency status, MCAT scores, and undergraduate GPA, for a total of 15 independent variables.^2^ In the second model, we estimated OLS models to predict changes in CR visits 21 days after the exam (the dependent variable) as a function of changes in exam scores between those exams (the independent variable), after controlling for the aforementioned demographic and baseline undergraduate academic variables. The primary output of these models was linear regression coefficients, though the models were also used to generate predictions in changes in exam scores and recreation use. As mentioned above, these changes in CR use or academic performance were between exam periods, which were typically 2–3 weeks apart from each other. Stata MP 11.2 was used to carry out these analyses.

## Results

### Descriptive analysis

**Fig. 1:**
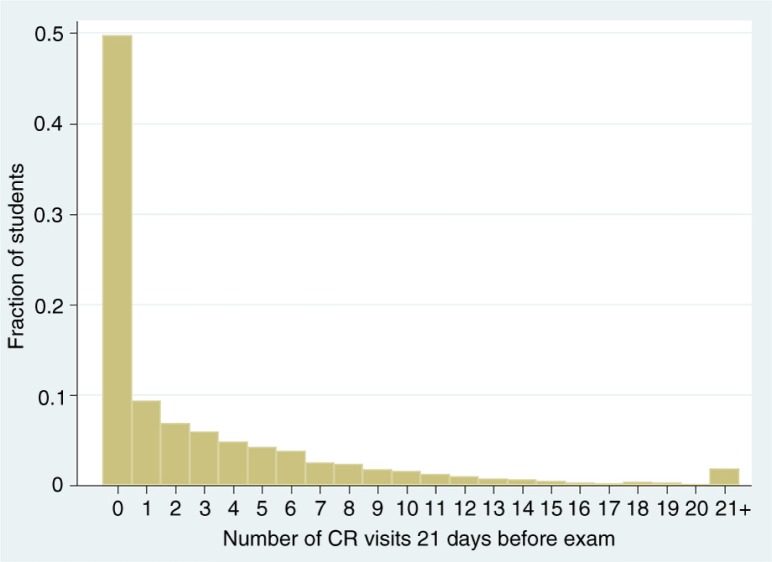
Distribution of number of CR visits 21 days before an exam. The distribution was derived from approximately 20 exam periods each for 408 first-year medical students across four cohorts from 2006 to 2010.

Table 1 shows both baseline and time-varying factors of the 408 traditional first-year medical students that we consider in our analyses. Our sample is similar to other public medical schools in the United States. Overall, the average age at matriculation was 24. About 44% of students reported being female, and almost 71% were in-state residents. Further, the class was ethnically heterogeneous, with 30% reported being of Asian descent, 10% Hispanic, and about 6% African American, with the remaining 49% being classified as Caucasian. Students presented to the medical school with strong academic profiles at the time of admission, with an average undergraduate cumulative GPA of 3.56 and MCAT scores around 10 for Biological and Physical Sciences, with a slightly lower average Verbal Reasoning MCAT score. Turning to the frequency of CR attendance (Fig. 1), though students on average used CR facilities approximately once per week, its distribution was highly skewed. Approximately half of students (an average of 204 students during each exam date) did not attend a CR facility at all in the 3 weeks prior to an exam date; approximately 10% of students (an average of 38 students during each exam date) used it only once in this 21-day window. However, one-third of individuals (an average of 138 students) used the CR facilities at least three times in the 21-day period (an average of one visit per week), implying that a sizeable fraction of the students used the facilities regularly. It is important to note that many of the individuals who did not use the CR facilities at all during the 3-week period prior to the exam chose not to use the facilities at all throughout the year. They may have, for instance, used an additional recreation facility or alternatively, did not exercise at all. Given that our analysis relied on changes in levels of recreation use to predict changes in exam scores, many of these non-CR attending students did not contribute variation to this variable, and were therefore effectively omitted from these models.

**Table 1:** Characteristics of first-year medical students (*N*=408)

	N (%) or Mean (Std. Dev)
Changes in exam scores and CR visits between exam dates	
ΔExam score (percentage points)	0.16 (0.12)
ΔCR visits 21 days before exam	−0.08 (0.02)
ΔCR visits 21 days after exam	0.01 (0.02)
Demographic and baseline academic characteristics	
Asian	122 (29.9%)
African American	24 (5.9%)
Hispanic	42 (10.3%)
Caucasian	200 (49%)
Missing race or ethnicity	20 (4.9%)
Male	231 (56.6%)
In-state resident	289 (70.8%)
Age at matriculation	23.95 (2.62)
Undergraduate GPA	3.56 (0.35)
MCAT Score–Biological Sciences	10.18 (1.62)
MCAT Score–Physical Sciences	9.95 (1.86)
MCAT Score–Verbal Reasoning	9.34 (1.34)

ΔExam score refers to changes in aggregate exam score from one exam date to the next, which is usually in 2–3 weeks (though exam dates can be closer together). Changes in exam scores are interpreted as percentage point differences between the scores on one exam date and the next. ΔCR visits refers to the changes in CR visits 21 days before or after an exam between one exam date and the next.

**Fig. 2:**
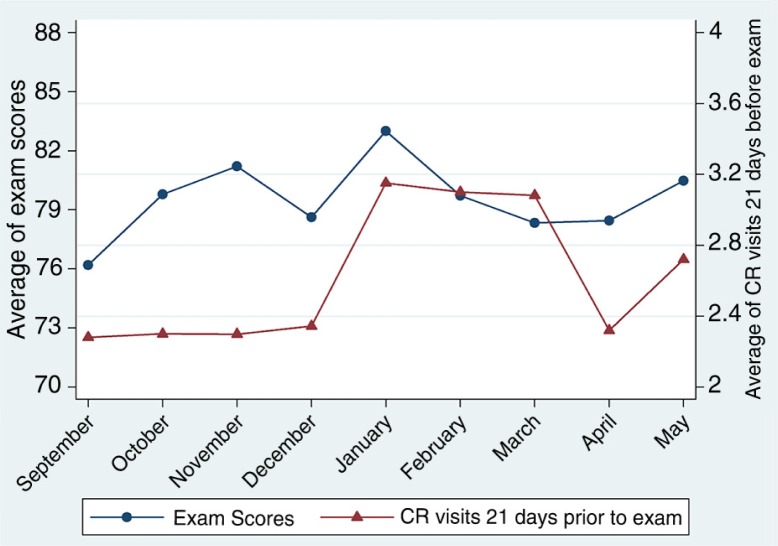
Average exam scores and CR visits by month of the academic year. Each point corresponds to the mean percentage exam score (in blue), and number of campus recreation (CR) visits (in red) for 408 first-year medical students for exams given in each month of the academic year. Number of CR visits was calculated as the number of times a student visited a CR facility in the 21-day period prior to an exam date.

Figure 2 shows exam performance and CR by month of the academic year. Exam performance was relatively constant throughout the year, with the average exam score for M-1 students falling between 76% and 84% throughout the year. Students performed slightly better overall later in the academic year (January through May), with an average exam score of about 80%, compared to earlier in the year (August through December), when exam scores averaged slightly over 78%. In terms of participation in CR activities, first-year students visited CR facilities between 2 and 3 times in the 21-day span before an examination. This corresponds to slightly less than one visit per week throughout the academic year. The changes in both CR use and exam performance from one exam date to the next, however, are rather mild; exam scores improve, on average by 0.16 percentage points, whereas the average number of CR visits 21 days after an exam essentially does not change. These values represent the average net change for all students across the year; indeed, individual students’ test scores and CR visits tend to fluctuate somewhat throughout the year (see Fig. 2), which allows for the estimation of our models.

### The role of exam performance on subsequent CR use

**Fig. 3:**
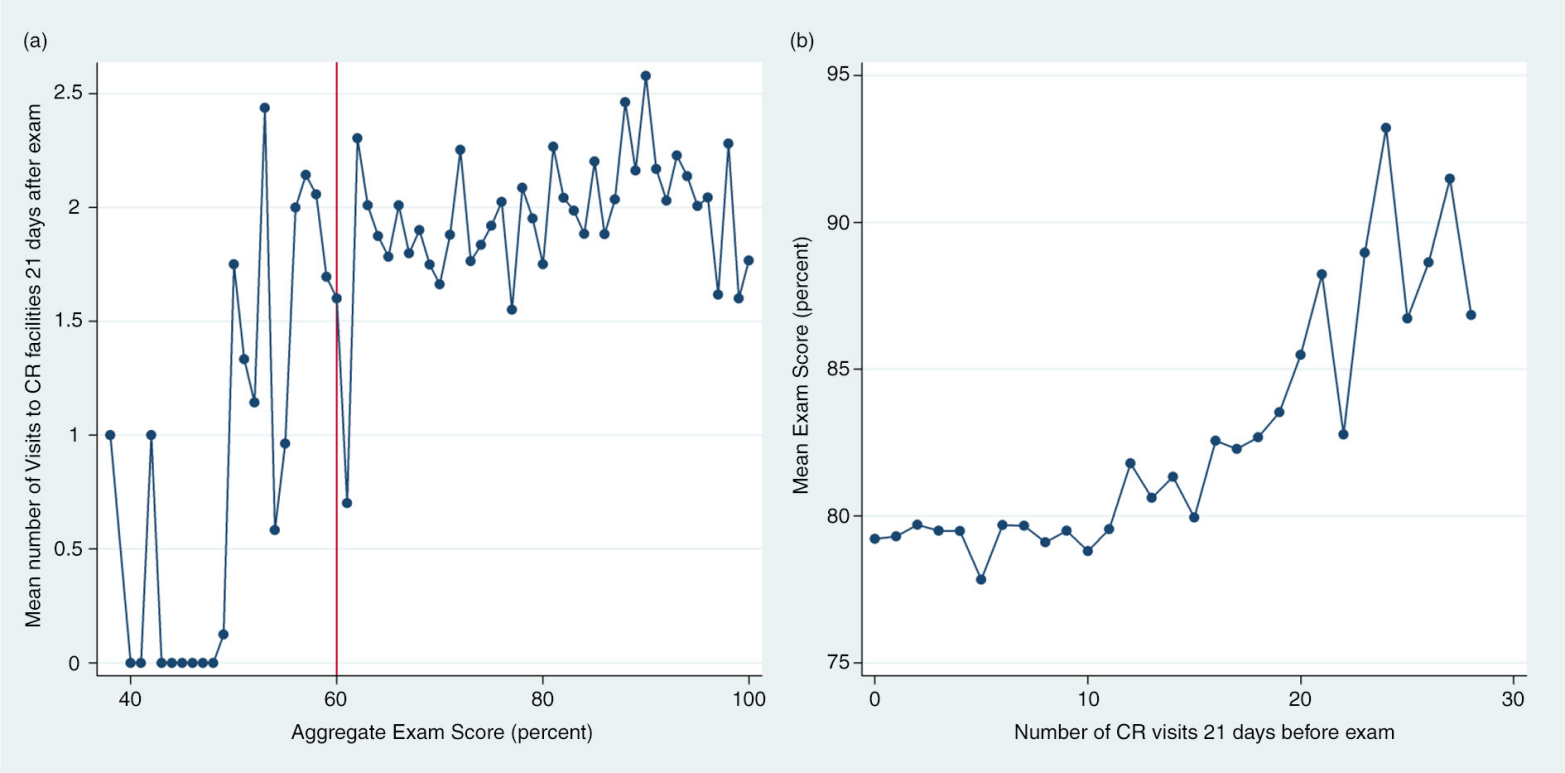
Conditional means of CR visits and exam performance. An exam score of 60% represents the passing level for most exams. The data are based on approximately 20 exam sessions each for 408 first-year medical over 4 years. In (a), the number of CR visits is calculated as the number of times a student visited a CR facility in the 21-day period after an exam date. In (b), the number of CR visits is calculated as the number of times a student visited a CR facility in the 21-day period prior to an exam date.

We estimated several regression models to ascertain the reciprocal relationship changes in academic performance and changes in CR utilization. We begin by exploring the role of exam performance on CR utilization in first-year medical students. Figure 3a presents descriptive means of CR visits in the 21 days after an exam by the numeral exam score. The figure suggests that individuals who are struggling to achieve a passing score on their exams (generally 60%) tend to have very low CR utilization levels; especially so for those individuals scoring 50% or less on their exams. Table 2, Column 1 presents linear regression results that describe the role of changes in exam performance from one exam date to the next on changes in the number of CR visits 21 days after an exam. The regression coefficient of interest suggests that an individual will increase their attendance at the CR facilities by 0.007 in 21 days if their exam score increases by a single percentage point. The increase in CR attendance, while statistically significant, is a practically negligible amount. However, changes in CR attendance may become more meaningful with large fluctuations in exam performance. In particular, individuals who have large drops in their exam performance might have a tendency to use the CR facilities somewhat less.

**Table 2:** Results of Ordinary Least Squares (OLS) regression models estimating the reciprocal relationship between changes in academic performance and changes in CR use

	(1)		(2)	
	
	ΔCR visits 21 days after exam		ΔExam score	
	
	Beta	Std. error	Beta	Std. error
ΔExam score	0.007***	0.002		
ΔCR visits 21 days before exam			0.180**	0.079
Asian	0.025	0.023	−0.052	0.073
African American	0.088**	0.037	0.220	0.153
Hispanic	0.059**	0.029	−0.053	0.103
Missing race or ethnicity	−0.017	0.061	0.037	0.098
Male	0.000	0.017	0.031	0.061
In-state resident	−0.001	0.024	0.032	0.067
Age at matriculation	0.003	0.002	0.022**	0.011
Undergraduate GPA	0.015	0.027	−0.016	0.097
MCAT Score–Biological Sciences	0.005	0.008	−0.021	0.023
MCAT Score–Physical Sciences	−0.007	0.008	−0.022	0.021
MCAT Score–Verbal Reasoning	0.003	0.005	0.001	0.016

Caucasian is the reference category for race and ethnicity. Year indicator variables are included in both models.

CR: campus recreation.

*** *p*<0.01

* *p*<0.05.

**Fig. 4:**
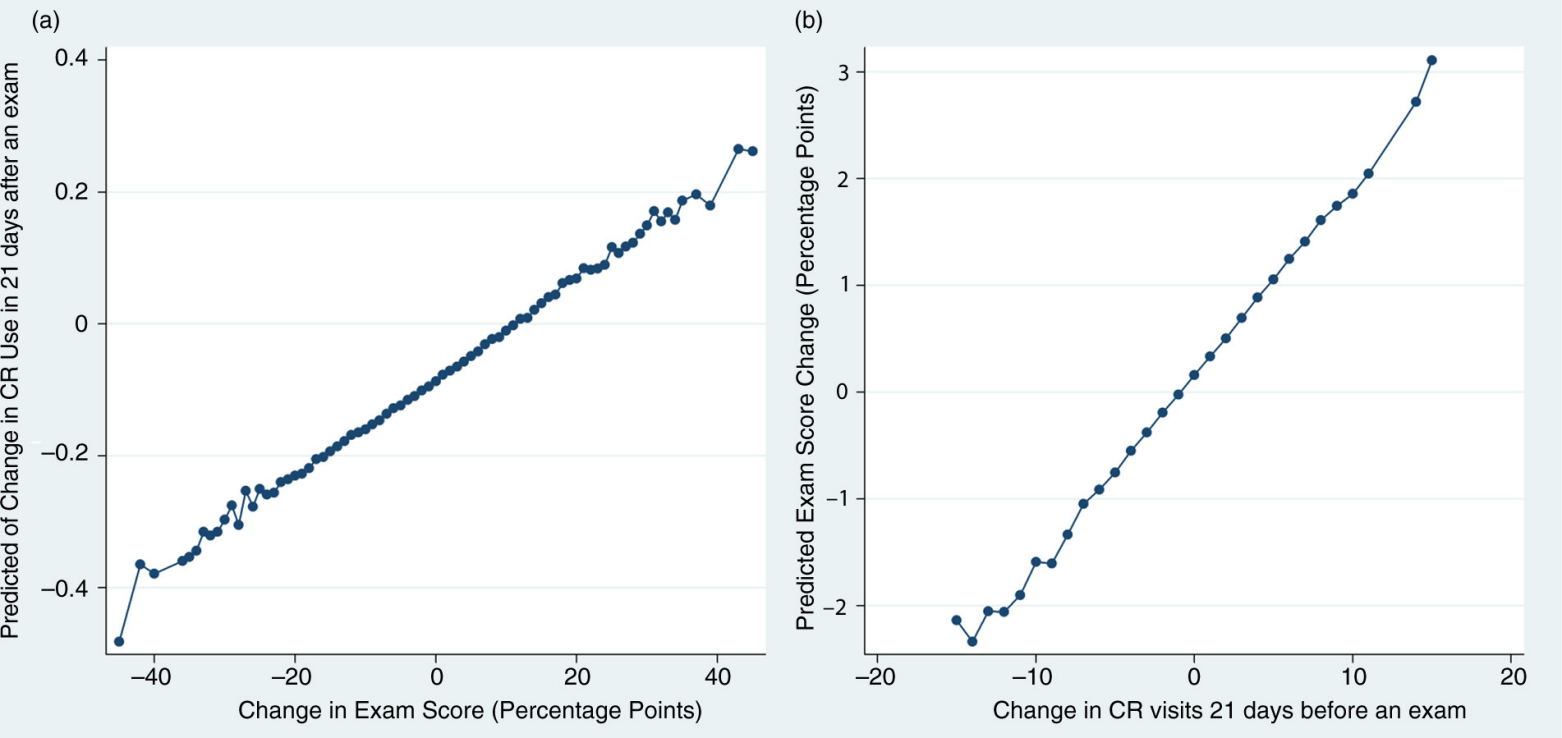
Predicted changes in CR use and academic performance from regression models. Graph (a) plots the average predicted values of ΔCR visits from the regression shown in Column 1 of Table 2. Graph (b) plots the average predicted values of ΔExam Score from the regression shown in Column 2 of Table 2.

The results of this regression model are visualized in Figure 4a. In this figure, the outcome variable of interest (changes in CR visits) was predicted and averaged across individuals, and plotted by changes in examination score. This plot suggests that an individual who increases her exam score by 20 points (which would represent a huge improvement for almost any student) would be predicted to increase her CR attendance by about 0.05 times. However, if she decreased her exam score by 20 points, the predicted decrease in her CR attendance is about 0.25 in a 21-day span, which approximates to one visit during a semester.

With the exception of race and ethnicity, other demographic and educational variables were largely insignificant in predicting changes in CR use, including MCAT score and undergraduate GPA.

### The role of CR use on exam performance

The role of changes of CR facilities in predicting changes in academic performance was assessed in a similar manner to the analysis in the reverse direction (discussed above). Figure 3b shows the mean exam performance based on the number of CR visits completed in a 3-week period before an exam. Overall, there was a net upward trajectory when a student increased their number of CR visits. This is especially true among individuals who used CR facilities about once daily (20 visits and above), who had substantially increased exam scores. These students had an 8.3 percentage point higher average exam score than those who use CR facilities 19 times or less (*p*<0.01). However, these very high scores are driven by observations from a relatively small number of individuals, who consistently used CR facilities at least daily.

Results from linear regression models are presented in Column 2 of Table 2, with changes in exam scores being predicted as a function of changes in CR visits. Overall, the results suggest that if an individual changes their CR use by one visit, their exam score would increase by 0.18 percentage points. Put another way, an individual who increases their CR visits by five would be predicted to increase their exam score by about 1 percentage point. While an increase of five CR visits in a 21-day period may seem large, it is plausible for someone who attends CR facilities infrequently to begin attending 2–3 times per week, which translates to 6–9 times in a 3-week period. Further, as some exam dates can contain 100 multiple-choice items or more, an increase in 1 or 2 percentage points may not be trivial.

Figure 4b presents the results of this regression visually, which suggests that individuals who make large increases in their visits to CR facilities (10 or more visits in a 21-day period) on average, are likely to boost their performance on exams by an average of 2–3 percentage points. Likewise, individuals who lower their number of visits significantly, on average, lower their exam performance by up to 2 percentage points. This finding has strong implications, as students may choose to lower their levels of CR use if they perceive their exam performance to be suffering, possibly in efforts to increase their study time. Our result suggests that this strategy may, in fact, do these students more harm than good.

Other demographic and baseline academic factors were insignificant in these models, though age at matriculation was marginally associated with increased exam performance.

## Discussion

Medical student stress is a growing concern within United States medical education. This concern is justified not only from a psychological perspective but also an academic one as students with higher levels of stress are more likely to develop academic deficiencies, which may ultimately have negative consequences on patient care. Previous work has also suggested that personal health habits, such as physical exercise, tend to be compromised as the academic year progresses and as stress continues to mount. However, no studies have investigated the relationship between academic performance and frequency of visits to athletic facilities among medical students.

Our descriptive analysis found that individuals who do poorly on examinations, on average, tend to use CR facilities less, whereas those who use CR facilities very often (20 or more visits in a 21-day span), on average, tend to do better on exams. Not surprisingly, both CR use and academic performance are highly persistent, as evidenced by the small changes in both CR visits and academic performance presented in Table 1. This may explain, in part, why MCAT scores or undergraduate GPA was significantly associated with future academic performance, or why with demographic and baseline characteristics were not associated with changes in future CR use in the linear regression models.

In considering the reciprocal associations of CR use on academic performance and vice versa, increased CR use has a positive association with subsequent exam scores, with an increase of five CR visits in a 21 day period corresponding to an increase in exam scores of about 1 percentage point. Similarly, those who decreased their CR visits by five CR visits were likely to decrease their exam score by about 1 percentage point. The results of our analyses suggest that some students who perform poorly on an examination (e.g., fail) might tend to stop visiting CR facilities in an effort to devote more time to studying and potentially improve their exam scores. Our findings largely contradict this logic, which suggest that individuals may actually decrease their performance by following this strategy. If a struggling student were to stop using CR facilities in order to devote more time to studying, this could potentially hurt the students by (on average) decreasing exam scores, by compromising a healthy lifestyle, which can set the foundation for good habits in later years of medical school, during residency training, and ultimately, as practicing physicians.

Turning to the relationship between academic performance and the frequency of CR use, we find that changes in exam performance are positively associated with subsequent CR use. This implies that individuals with increases in exam performance are more likely to increase their usage of CR facilities more frequently by the next exam date. There are several explanations that may justify this positive association. First, individuals who are having higher levels of academic performance may have less ‘pressure’ to pass their classes and therefore more available time to allocate to other activities, such as visiting recreation facilities. Likewise, higher-achieving students may possess better time-management skills that enable them to perform better in classes, as well as to attend CR facilities more frequently. Promoting positive health behaviors, including exercise, among medical students is a concern among medical schools throughout the country. Individuals with lower academic performance may be a group that is particularly vulnerable to lapses in these activities, and as such can especially benefit from programs aimed at encouraging these behaviors. Further studies can elucidate the precise mechanisms, such as increased time efficiency, improved mental health, and social connections, by which the positive correlation exists between CR use and academic performance.

Our study has several limitations. As discussed earlier, our measure of CR use is not a direct measure of exercise. While we believe that most medical students use CR for exercise, it is possible that some students utilize the facilities for other purposes. Additionally, even though the fees required to use CR facilities are required of all students, individuals may choose to exercise in other settings, including private fitness clubs or at home. We also only collected our data from a single state medical institution in Illinois; that is, our findings might not reflect the relationship between academic performance and recreation use among medical students in different geographical locations and in private settings. Additionally, there are slight changes to the overall makeup of the students and curriculum (e.g., faculty turnover and testing schedule changes) that change annually, and our findings may have differed were the data collected more recently. Finally, given different training models and exam schedules across different institutions and training programs, it is difficult to generalize these findings to other types of students (including those in other health professions).

## Footnotes

1 The College also enrolls students who repeat the first year, or who only partially participate in the curriculum as a result of decompression of the first year, or participate in the Medical Scholars Program (MSP, the MD/PhD program at the University of Illinois). This analysis excludes repeating, decompressed or Medical Scholars students, and focuses on full-curriculum students, which is our population of interest.

## Footnotes

2 Note that certain categorical variables, such as ethnicity and academic year, are transformed into multiple dichotomous variables.
